# Clusterin and Its Potential Regulatory microRNAs as a Part of Secretome for the Diagnosis of Abnormally Invasive Placenta: Accreta, Increta, and Percreta Cases

**DOI:** 10.3390/life11040270

**Published:** 2021-03-24

**Authors:** Angelika V. Timofeeva, Ivan S. Fedorov, Mariya M. Pirogova, Oksana N. Vasilchenko, Vitaliy V. Chagovets, Larisa S. Ezhova, Tatiana M. Zabelina, Roman G. Shmakov, Gennadiy T. Sukhikh

**Affiliations:** 1 Kulakov National Medical Research Center of Obstetrics, Gynecology, and Perinatology, Ministry of Health of Russia, Ac. Oparina 4, 117997 Moscow, Russia; is_fedorov@oparina4.ru (I.S.F.); mm_pirogova@oparina4.ru (M.M.P.); o_vasilchenko@oparina4.ru (O.N.V.); v_chagovets@oparina4.ru (V.V.C.); l_ezhova@oparina4.ru (L.S.E.); t_zabelina@oparina4.ru (T.M.Z.); r_shmakov@oparina4.ru (R.G.S.); g_sukhikh@oparina4.ru (G.T.S.); 2 Department of Obstetrics, Gynecology, Perinatology and Reproductology, First Moscow State Medical University Named after I.M. Sechenov, 119991 Moscow, Russia

**Keywords:** pregnant women, miRNA, abnormally invasive placenta, accreta, increta, percreta, RT-PCR, NGS, peripheral blood plasma, secretory clusterin, soluble E-cadherin

## Abstract

Magnetic resonance imaging (MRI) and ultrasound methods used for the diagnosis of an abnormally invasive placenta (AIP) have a wide range of sensitivity (Se, 33–93%) and specificity (Sp, 71–100%) levels, which results in a high risk of unfavorable maternal and perinatal outcomes. The relevance of optimizing the diagnosis of AIP is beyond doubt. Given the epigenetic nature of trophoblast invasion, we aimed to quantitate microRNAs and proteins of their target genes that are potentially associated with AIP in blood plasma samples from 64 pregnant women at gestation weeks 30–34 by reverse transcription coupled with polymerase chain reaction (RT-PCR) and Western blotting, respectively. Statistically significant increases in the expression levels of hsa-miR-17-5p, hsa-miR-21-5p, hsa-miR-25-3p, hsa-miR-92a-3p, and hsa-miR-320a-3p were revealed in the groups of women with AIP (accreta, increta, percreta) relative to the group of women with scars on the uterus or to the group with placenta previa. Opposite changes in the expression level of “gene–target protein/miRNA” pairs were found for the α-subunit of the clusterin secretory form and any of the hsa-miR-21-5p, hsa-miR-25-3p, hsa-miR-92a-3p, hsa-miR-320a-3p, and hsa-miR-17-5p in all cases of AIP. The developed logistic regression models to diagnose AIP cases of various severity gave Se values of 88.8–100% and Sp values of 91.6–100% using a combination of hsa-miR-21-5p, hsa-miR-92a-3p, hsa-miR-320a-3p, or clusterin levels.

## 1. Introduction

Abnormal uterine scars after a previous cesarean section, especially in combination with the occurrence of placenta previa, are thought to serve as a basis for defective decidualization and deep trophoblast invasion [1]. Many research groups are working to understand the molecular biological mechanisms related to abnormally invasive placenta (AIP) pathogenesis and associate it with the formation of (i) a placenta with a proangiogenic phenotype (decreased expression level of antiangiogenic sFlt-1 and increased levels of angiogenic growth factors (vascular endothelial growth factor, angiopoietin-2) [2]; (ii) increased proliferation of extravillous trophoblast cells, supported by both syncytiotrophoblast (increased expression levels of epidermal growth factor receptor and c-erbB-2 oncogene) and impaired regulation of apoptosis in the extravillous trophoblast cells themselves (decreased level of INSL4 expression, inducing apoptosis) [3,4]; (iii) overexpression of matrix metalloproteinases in the placenta (MMP2, MMP9), which ensures the migration of trophoblast cells by reorganizing the extracellular matrix [5]; (iv) increased expression of pregnancy-associated plasma protein A in the syncytiotrophoblast, providing an increased local concentration of insulin-like growth factor, which controls the uptake and transport of glucose and amino acids in trophoblast cells and their invasion into the decidual tissue [6,7,8]; and (v) an abnormally aggressive epithelial–mesenchymal transition in the extravillous cells of the trophoblast, which ensures their high invasiveness and does not stop by the beginning of the second trimester but proceeds throughout pregnancy [9].

Some of the main regulators of biological processes, including those mentioned above, are small noncoding RNAs, in particular, miRNAs [10,11,12]. Regulation by miRNA molecules is based on RNA interference and occurs at the transcriptional and post-transcriptional levels, namely (i) as a part of the RNA-induced silencing complex, microRNA recognizes the sequence of the target RNA, and the endonuclease activity of Argonaute proteins leads to the cleavage of the target RNA, and (ii) protein synthesis is blocked by the interaction of the RISC complex with translation initiation factors. miRNAs may participate in the epigenetic silencing of genes by DNA methylation, either indirectly through binding to mRNAs encoding transcription factors or by directly affecting the mRNAs coding for DNA methyltransferases [13]. In turn, the activity of promoters of some miRNAs depends on the expression level of DNA methyltransferases, which form regulatory feedback loops [14]. According to the base miRWalk, miRanda, RNA22, and Targetscan, one miRNA molecule regulates the expression level of tens to hundreds of genes, and at the same time, one gene is a target for many miRNAs. Nevertheless, as regulator of mRNA, miRNA itself can be regulated by pseudogene transcripts, circRNAs, viral RNAs, and lncRNAs through sequestration mechanism [15].

In light of the above, it is clear that the pathogenesis of AIP is complex. Therefore, to diagnose this condition, it is sufficient to identify molecules that reflect components of pathogenesis and are secreted into the biological fluids of the body.

In the present study, we focused on the miRNAs that potentially regulate the epithelial–mesenchymal transition (EMT), which determines the ability of extravillous cytotrophoblast cells to invade. The EMT program is triggered by growth factors synthesized in stromal cells and the activation of various signaling pathways: transforming growth factor beta/bone morphogenetic protein, WNT/β-catenin, Hedgehog, Notch, and phosphatidylinositol 3-kinase /AKT [16]. Most prominent among these cells are macrophages and activated resident fibroblasts that accumulate in chronically inflamed tissues as a result of injury and release growth factors, chemokines, MMP-2, MMP-3, and MMP-9 [17]. This may be the case in chronic endometritis after repeated curettage of the uterine cavity, leading to morphological disorders of the decidual layer of the uterus and being a possible cause of AIP [18]. The result of the activation of these signaling pathways is the expression of the transcriptional repressors of epithelial genes, in particular E-cadherin, and as a consequence, the cell molecular phenotype changes from epithelial to mesenchymal, providing restructuring of the actin cytoskeleton, decreased intercellular adhesion contacts, and increased cell proliferation and migration. In addition, the degree of proteolysis of E-cadherin, which determines the formation of its soluble form, affects the invasiveness of cells, in particular, trophoblast cells. It has been shown that with pathological invasion of the placenta, the process of E-cadherin proteolysis is disrupted, which is probably an important molecular mechanism for controlling the invasiveness of the trophoblast during placenta accreta [19]. Thus, assessment of the soluble form of E-cadherin in the blood plasma of pregnant women could be a useful way to evaluate the risk of AIP. 

Another key molecule related to the regulation of cell phenotype transformation is clusterin [20,21]. Clusterin is constitutively expressed in almost all tissues and is present in all organismal body fluids, albeit at varying levels dependent on cell type and physiological status [21]. From the initial protein precursor, different isoforms with various functions can generate: a nuclear isoform (49 kDa), the cytosol and mitochondria isoform (53 kDa), as well as glycosylated proteins that are cleavaged in the endoplasmic reticulum/Golgi apparatus and secreted in the form of α-β heterodimer (75−80 kDa) [22,23,24]. A change in the balance between the isoforms of clusterin is tightly controlled in the cell during different steps of tumor progression associated with changes in apoptotic, proliferative, and invasive processes [25,26,27].

Clusterin integrates multiple signaling pathways involved in tumorigenesis and the progression of various malignancies, among which are those involved in epithelial mesenchymal transition such as IGF-1, TGFβ, VEGF, AKT/MAPK, and Wnt signaling [21]. The expression regulation of clusterin different forms is under control of the tumor–microenvironment interactions providing local cancer growth, invasion, and metastases [28]. Similar mechanisms for controlling the level of clusterin expression can take place during the invasion of embryonic trophoblast cells into the decidual layer of the endometrium. In normal pregnancy, the complex embryo–endometrium crosstalk involving numerous cytokines, growth factors, receptors, and mediators, is necessary [29] to ensure a strictly defined degree of invasion of the trophoblast cells into the decidual layer, without affecting the deeper layers of the endometrium and the adjacent myometrium. Most likely, morphological changes in the decidua due to chronic inflammation in the uterus lead to pathological invasiveness of the placenta after embryo implantation as a result of impaired expression level regulation of clusterin.

The specificity of clusterin expression for syncytiotrophoblast cells and the endothelium of chorionic villi has been proven, and increased expression has been found in preeclampsia [30,31], the key pathogenetic mechanism of which is the dysregulation of the phenotypic transformation of trophoblast cells and their reduced invasive ability. However, there are still no data on changes in the expression and role of clusterin in the development of AIP.

In connection with the above, we aimed to quantify soluble E-cadherin and the secreted form of clusterin as well as the role of miRNAs as potential regulatory molecules in the expression levels of these compounds in the blood plasma of pregnant women with AIP of various levels of severity to assess the diagnostic value of each of them for use in clinical practice.

## 2. Materials and Methods

### 2.1. Patients

In total, 64 pregnant women aged between 18 and 45 years with cesarean section indications were enrolled in the study and comprised the five groups (Table 1). The inclusion criteria for the study were singleton pregnancy, scar on the uterus after previous cesarean section or myomectomy, placenta previa, and pathological invasion of the placenta (placenta accreta, increta, percreta). The exclusion criteria from the study were preeclampsia and acute phase or exacerbation of infectious and inflammatory diseases. Pathological invasion of the placenta was diagnosed by ultrasound and magnetic resonance imaging (MRI) studies carried out at 30–34 weeks of pregnancy, during which time peripheral blood was sampled. The pregnant women delivered their babies by cesarean section, and the diagnosis of pathological invasion of the placenta was confirmed by histological analysis of the material of the resected uterine wall with the area of pathological invasion of the placenta. Written informed consent was obtained from each patient, and the study was approved by the ethics committee of the National Medical Research Center for Obstetrics, Gynecology, and Perinatology, named after Academician V.I. Kulakov of Ministry of Healthcare of the Russian Federation (protocol No 8, approval date: 31 October 2019).

### 2.2. RNA Isolation from Peripheral Blood Plasma

Venous blood samples from pregnant women were collected into S-MONOVETTE tubes containing EDTA KE (Sarstedt AG&Co., Nümbrecht, Germany, cat. No 04.1915.100), centrifuged for 20 min at 300× *g* (4 °C) followed by plasma collection and recentrifugation for 10 min at 14,500× *g*. RNA was extracted from 200 µL of blood plasma using an miRNeasy Serum/Plasma Kit (Qiagen, Hilden, Germany, cat. No 217184).

### 2.3. RNA Isolation from the Placental Tissue

Placental tissue samples were collected for study no later than 10 min after delivery. A 5 mm-thick tissue slice was collected by passing only through the maternal surface of a placenta free of myometrium directly in the area of pathological invasion (P-area) and outside this area (N-area), and this was immediately frozen in liquid nitrogen for subsequent storage at −80 °C. Total RNA was extracted from 20–40 mg of placental tissue using an miRNeasy Micro Kit (Qiagen, Hilden, Germany, catalog No. 217084), followed by an RNeasy MinElute Cleanup Kit (Qiagen, Hilden, Germany, catalog No. 74204). The RNA concentration was measured using a Qubit fluorometer 3.0 (Life Technologies, Singapore, cat. Q33216). The sample quality of the total RNA was examined on an Agilent Bioanalyzer 2100 (Agilent, Waldbronn, Germany, cat. No G2939A) using an RNA 6000 Nano Kit (Agilent Technologies, Vilnius, Lithuania, cat. No. 5067-1511). Total RNA samples with an RNA integrity number (RIN) of at least 8 were used for further study.

### 2.4. Reverse Transcription and Quantitative Real-Time PCR

Seven microliters from 14 µL of total RNA column eluate (miRNeasy Serum/Plasma Kit, Qiagen, Hilden, Germany, cat. No 217184) extracted from 200 µL of blood plasma was converted into cDNA in a reaction mixture (20 µL) containing 1x Hispec buffer, 1x Nucleics mix, and miScript RT in accordance with the miScript^®^ II RT Kit protocol (Qiagen, Germany, cat. No 218161); then, the sample volume was adjusted with deionized water to 200 µL. The synthesized cDNA (2 µL) was used as a template for real-time PCR using a forward primer specific for the studied RNA (Table 2) and the miScript SYBR Green PCR Kit (Qiagen, Germany, cat. No 218075). The following PCR conditions were used: (1) 15 min at 95 °C and (2) 40 cycles at 94 °C for 15 s, an optimized annealing temperature (52–60 °C) for 30 s and 70 °C at 30 s in a StepOnePlus^TM^ thermocycler (Applied Biosystems, Waltham, MA, USA, cat. No 4376600). The relative expression of miRNA in the blood plasma sample was determined by the ∆Ct method using hsa-miR-382-5p (MIMAT0000737, miRBase, available online: http://www.mirbase.org/, accessed on 24 March 2021) as the reference RNA.

### 2.5. miRNA Deep Sequencing

cDNA libraries were synthesized using 500 ng of total RNA from the placenta using a NEBNext^®^ Multiplex Small RNA Library Prep Set for Illumina^®^ (Set11 and Set2, New England Biolab^®^, Frankfurt am Main, Germany, cat. No E7300S, E7580S), amplified for 14 and 18 PCR cycles, respectively, and sequenced on a NextSeq 500 platform (Illumina, San Diego, CA, USA, cat. No SY-415-1001). The adapters were removed with Cutadapt. All trimmed reads shorter than 16 bp and longer than 30 bp were filtered, and only reads with a mean quality higher than 15 were retained. The remaining reads were mapped to the GRCh38.p15 human genome and miRBase v21 with the bowtie aligner [32]. Aligned reads were counted with the featureCount tool from the Subread package [33] and with the fracOverlap 0.9 option, so the whole read was forced to have a 90% intersection with sncRNA features. Differential expression analysis of the sncRNA count data was performed with the DESeq2 package [34].

### 2.6. Western Blotting

Eleven micrograms of the total protein of the plasma sample, the concentration of which was determined by the Biuret method, was (i) denatured at 65 °C for 5 min in a buffer containing 50 mM tris(hydroxymethyl)aminomethane (Sigma-Aldrich, St. Louis, MO, USA, cat. No T4661) hydrochloride (Tris-HCl) at a pH of 6.8, 1% sodium dodecyl sulfate (SDS) (VWR Life Science AMRESCO, Framingham, MA, USA, cat. No Am-O227-0.1), and 10% glycerol (AppliChem, Darmstadt, Germany, A4443), 238 mM 2-mercaptoethanol (VWR Life Science AMRESCO, Framingham, MA USA, cat. No Am-O482-0.1); (ii) separated in 10% SDS-PAGE in Tris-tricine buffer (100 mM Tris, 100 mM tricine (Sigma-Aldrich, St. Louis, MO, USA, cat. No T0377), 0.1% SDS), as recommended by H. Schägger [35]; (iii) transferred to a PVDF membrane (0.45 µm, Immobilon, Merck Millipore Ltd., Germany, catalog No. IPVH07850) by semi-dry transfer in a Trans-blot^®^ SD semi-dry transfer cell (BioRad, Hercules, CA, USA, cat. No 1703940) using two buffer systems (anode buffer: 40 mM 3-cyclohexylamino-1-propanesulfonic acid (CAPS) (Sigma-Aldrich, USA, cat. No SW18805), 60 mM Tris, pH = 9.6, 15% ethanol; cathode buffer: 40 mM CAPS, 60 mM Tris, pH = 9.6, 0.1% SDS) followed by membrane blocking with 1% non-fat dry milk (M7409, Sigma-Aldrich), 0.1% Tween^®^ 20 (Sigma-Aldrich, USA, cat. No P1379), 50 mM Tris-HCl at a pH of 7.5 and 150 mM NaCl (AppliChem Panreac ITW Companies, Darmstadt, Germany, A1371); and (iv) incubated with primary mouse monoclonal antibodies to E-cadherin at a dilution of 1:400 (5F133, Santa Cruz Biotechnology, Dallas, TX, USA, cat. No sc-71007) or primary mice monoclonal antibodies to the alpha subunit of clusterin at a dilution of 1:400 (B-5, Santa Cruz Biotechnology, Dallas, TX, USA, cat. No sc-5289), followed by incubation with goat anti-mouse secondary polyclonal antibodies conjugated with horseradish peroxidase at a dilution of 1:1000 (R&D Systems, Minneapolis, MN, USA, cat. No HAF007). Protein bands were visualized by enhanced chemiluminescence (SuperSignal™ West Femto Maximum Sensitivity Substrate, ThermoScientific, Rockford, IL, USA, catalog No. 34096) in the ChemiDoc MP gel documentation system (BioRad, Hercules, CA, USA, cat. No 12003154). Densitometric analysis of the band intensity was done using ImageLab™ Software (version 6.0 build 25 Standard Edition, BioRad Laboratories, Inc., Hercules, CA, USA). The size of the analyzed protein was determined in the ImageLabTM program based on the electrophoretic mobility of the PageRulerTM Plus Prestained Protein Ladder (ThermoScientific, Waltham, MA, USA, catalog no. 26619) added to the same SDS-PAGE as the blood plasma samples.

### 2.7. Statistical Analysis of the Obtained Data

For statistical processing, we used scripts written in R language [33] and RStudio [36]. The correspondence of the analyzed parameters to the normal distribution law was assessed by the Shapiro–Wilk test. When the distribution of data was different from normal, the Mann–Whitney test for paired comparison was used, and data were described as the median (Me) and quartiles Q1 and Q3 in the format Me (Q1; Q3). To reduce the instance of a false positive, Bonferroni correction for multiple testing and Holm–Bonferroni test were used as described in [37]. Bonferroni correction was applied between compared groups and among studied molecules. To identify the relationships among categorical variables, chi-square tests were performed. Since both quantitative and qualitative characteristics were analyzed, a correlation analysis was performed using Spearman’s nonparametric correlation test. The 95% confidence interval for the correlation coefficient was determined using the Fisher transformation. The value of the threshold significance level (*p*) was taken as equal to 0.05. If the *p*-value was less than 0.001, then *p* was indicated in the format *p* < 0.001.

## 3. Results

### 3.1. Histological Analysis of the Placenta

**Figure 1:**
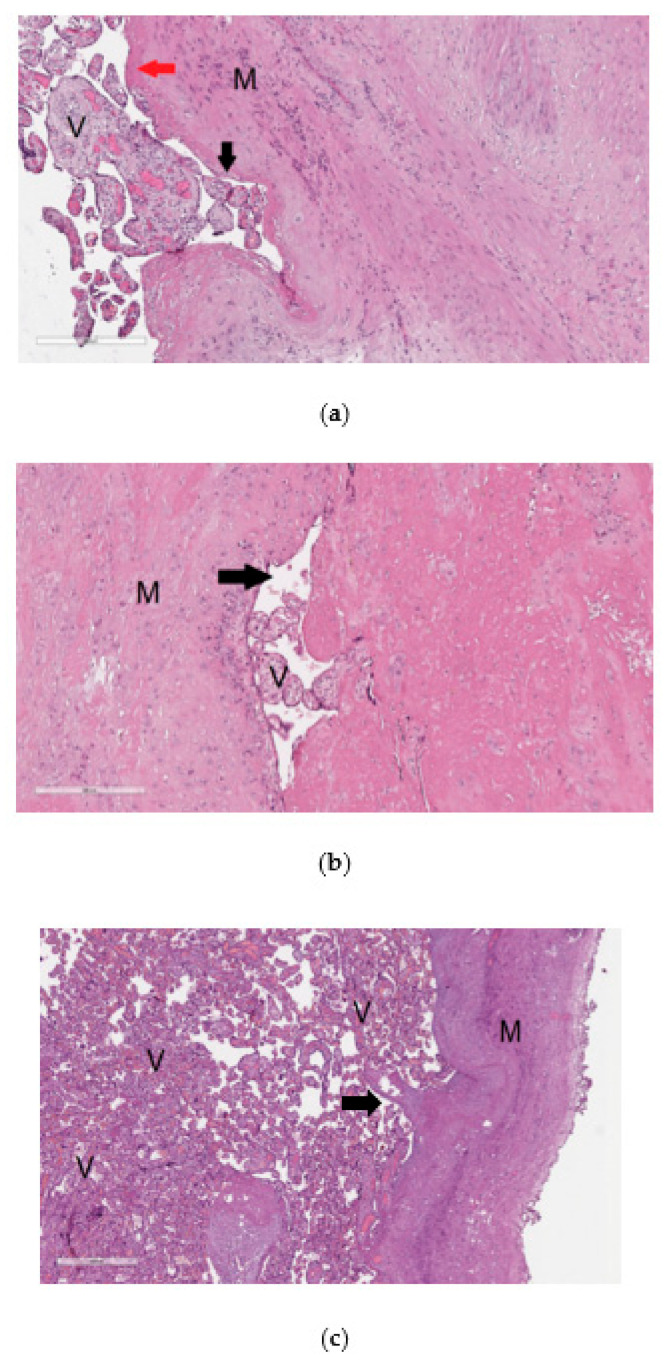
Histological analysis of the placenta with pathological invasion: placenta accreta (**a**), placenta increta (**b**), placenta percreta (**c**). In the images of histological sections (**a**–**c**), stained with hematoxylin and eosin, “V” denotes chorionic villi and ”M” denotes myometrium. In image (**a**) with 100× magnification, the mononuclear intermediate trophoblast is marked with a black arrow, and the fibrinoid is marked with a red arrow; in image (**b**) with 100× magnification and in image (**c**) with 40× magnification, the arrow indicates the lumen of the myometrium blood vessel.

The material from the resected uterine wall obtained after cesarean section was examined by histological analysis to reveal signs of pathological invasion of the placenta. The diagnosis of placenta accrete was based on changes in the content and ratio of normal histological components in the uterine wall, in particular, the absence or thinning of decidual tissue without visualization of placenta tissue infiltration into the myometrium (Figure 1a). Placenta increta (Figure 1b) and placenta percreta (Figure 1c) were characterized by prolapse of the chorionic villi into the walls of the large veins in the myometrium and the presence of chorionic villi among the muscle bundles up to the serous layer in the case of placenta percreta (Figure 1c).

### 3.2. Selection of miRNAs That Potentially Regulate E-Cadherin and Clusterin

According to four electronic databases—miRWalk, miRanda, RNA22, and Targetscan—hsa-miR-320a-3p, hsa-miR-17-5p, hsa-miR-21-5p, hsa-miR-1323, hsa-miR-25-3p, hsa-miR-138-5p, hsa-miR-34a-5p, and hsa-miR-92a-3p are the common potential regulators of the expression levels of E-cadherin and clusterin; hsa-miR-30a-5p and hsa-miR-30c-5p specifically bind to clusterin mRNA; and hsa-miR-371a-5p and hsa-miR-506-3p potentially regulate E-cadherin. However, it should be noticed that even with the existence of experimentally validated interactions between miRNA and mRNA of the target gene, as in the case of hsa-miR-92a-3p, hsa-miR-25-3p, hsa-miR-138-5p, and E-cadherin, or in the case of hsa-miR-21-5p, hsa-miR-17-5p, and clusterin according to the miRTargetLink Human database, the impact of miRNA on the mRNA of the target gene is not so unambiguous. This is due to the mechanisms of the regulation of miRNA activity, among which is the sequestration mechanism [15], along with post-translational modifications that impact target gene protein activity and function, among which are methylation, acetylation, glycosylation, phosphorylation, ubiquitination, and SUMOylation [38,39,40,41,42,43,44,45].

**Figure 2:**
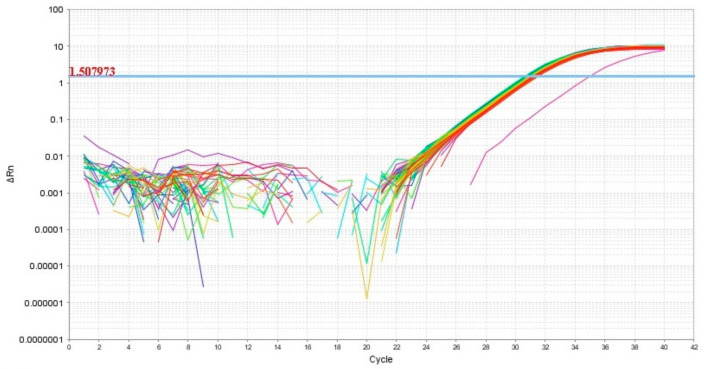
Amplification curves of hsa-miR-382-5p in 64 analyzed samples of blood plasma with indication of a threshold value, the point at which fluorescence reaches values above baseline levels.

Thus, in our study, 12 miRNAs were analyzed in 64 blood plasma samples from pregnant women from groups I–V (Table 1) by a quantitative real-time RT-PCR method using a sense primer specific for miRNA (Table 2) and a universal antisense primer from the miScript SYBR Green PCR kit. For data normalization, the use of miRNAs as normalizer seems to be more appropriate than other small noncoding RNAs such as RNU6, since this normalization strategy with the same family of RNA molecules comprises the same methods, such as extraction, reverse transcription, and PCR, and this conclusion is highlighted in the review article of Madadi S. et al. [46]. We found the expression level of hsa-miR-382-5p to be identical in all 64 analyzed samples except one (Figure 2). In this connection, the relative expression level of miRNAs in the blood plasma samples was determined by the ∆Ct method using hsa-miR-382-5p as the reference RNA.

### 3.3. Quantitative RT-PCR Analysis of the Selected miRNAs

**Figure 3:**
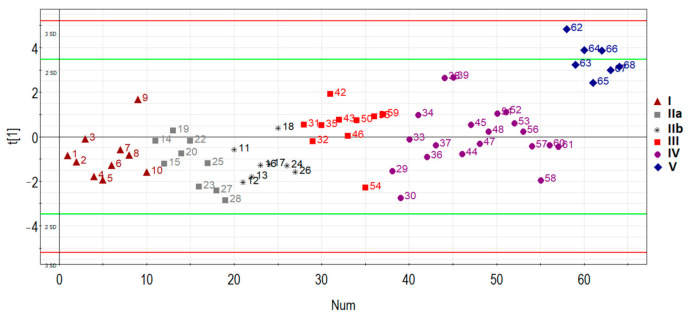
Principal component analysis (PCA) plot based on the miRNA dataset.

The principal component analysis was used to determine the clustering of patient samples based on the RT-PCR expression data of hsa-miR-17-5p, hsa-miR-21-5p, hsa-miR-25-3p, hsa-miR-92a-3p, and hsa-miR-320a-3p. In general, samples were clustered separately based on the disease status of the first principal component (Figure 3): patients with AIP (III, IV, V groups) clustered away from patients without AIP (I, IIa, IIb groups), except for several outliers in some groups which were removed for further analysis.

**Figure 4:**
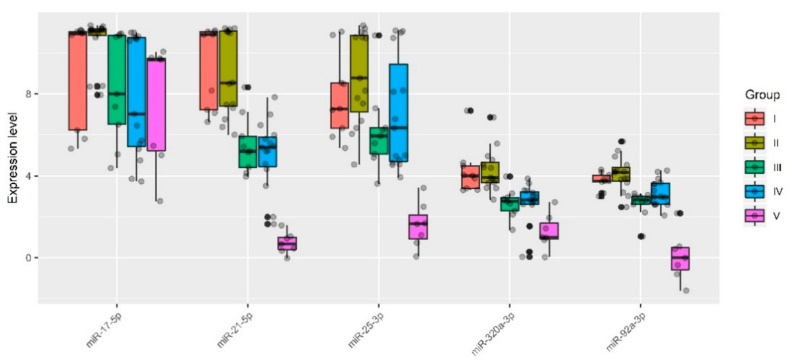
Quantitative RT-PCR data on the miRNA expression level (ΔCt values) in the peripheral blood plasma samples from pregnant women of groups I–V.

Specifically, one patient (ID = 9) was removed from group I (*n* = 10), one patient (ID = 19) was removed from group IIa (*n* = 9), one patient (ID = 18) was removed from group IIb (*n* = 8), one patient (ID = 54) was removed from group III (*n* = 10), four patients (ID = 29, 30, 36, 44, 58) were removed from group IV (*n* = 20), and none were removed from group V (*n* = 7). These samples were excluded for particular reasons. For example, according to MRI data of one particular patient (ID = 54), there was uterine wall swelling and deformation of its contour along with expansion of the veins of the placental bed and expansion of the lacunae of the maternal part of the placenta which indirectly indicated placenta accreta, although according to histological analysis, no areas of placenta accreta were found. As for the samples from patients (IDs = 29, 30, 36, 44, 58) from group IV, despite the diagnosis of “placenta increta” identified by MRI and ultrasound methods, a placental hernia was revealed during the cesarean section, the formation of which was not so much due to the pathological invasiveness of the placenta itself but rather the thinning of the myometrium and the divergence of its fibers. That is why miRNAs implicated in the EMT did not differentiate these samples from normal ones. The cohort of patients without outliers mentioned above was reanalyzed, and the results of pairwise comparison of the AIP groups with groups I or II are presented in Table 3, Table 4, Table 5 and Table 6. To visualize the data obtained, a box diagram was plotted (Figure 4).

Five out of 12 analyzed miRNAs (miR-17-5p, miR-21-5p, miR-25-3p, miR-92a-3p, and miR-320a-3p) showed statistically significant decreases in ΔCt median values in at least one of the groups with pathological invasion of the placenta (III–V) relative either to group I (Table 3 and Table 4) or to group II (Table 5 and Table 6). It is important to note that the lower the ΔCt value, the higher the microRNA expression level. There were no statistically significant differences in the expression levels of miR-17-5p, miR-21-5p, miR-25-3p, miR-92a-3p, and miR-320a-3p between groups I and II. However, the presence of placenta previa in group II and the absence of this condition in group I strongly influenced the statistical significance of differences in the expression level of some miRNAs when compared with groups III, IV, or V. For example, when taking into account the Bonferroni correction for multiple testing or applying the Holm–Bonferroni test, changes in the expression level of miR-21-5p and miR-320a-3p were significantly higher in groups III–V in comparison with both group I and group II; in contrast, miR-17-5p and miR-92a-3p were significantly upregulated in all groups with AIP (III–V) only in comparison with group II. As for miR-25-3p, the level of expression was significantly increased only in group V when compared to groups I or II. The variability in the significance of differences in groups III–V depending on the group selected for comparison (I or II) for some miRNAs may be due to the following reasons: i) the number of samples in each of the groups and/or ii) a slight change in the expression levels of these miRNAs in compared groups and/or iii) the presence of placenta previa in groups II–V and its absence in group I. From our point of view, it is more correct to compare each of groups III–V with group II and not with group I, since placenta previa possibly arises due to the characteristics of placental tissue itself, including the expression profile of genes encoding for the proteins and miRNAs that regulate them, and this parameter should be taken into account in all compared groups. Our assumptions are supported by literature data, in particular, by increased mRNA expression levels of HMGB1 and VEGF [47] and by decreased expression of β-catenin [48] in the placenta previa group compared to that in the normal group.

To assess the relationship between the microRNA expression level and the severity of AIP, the groups of analyzed samples were ranked in the following way: I < II < III < IV < V. The Spearman’s rank correlation method revealed the following statistically significant correlations: miR-17-5p (r = −0.33, *p* = 0.007), miR-21-5p (r = −0.68, *p* < 0.001), miR-25-3p (r = −0.36, *p* = 0.004), miR-92a-3p (r = −0.45, *p* < 0.001), miR-320a-3p (r = −0.55, *p* < 0.001). The revealed negative correlation between ΔCt miRNA and the severity of AIP indicated a positive correlation between increased expression levels of miR-17-5p, miR-21-5p, miR-25-3p, miR-92a-3p, and miR-320a-3p in the peripheral blood plasma of pregnant women and the severity of AIP.

### 3.4. Deep Sequencing of miRNAs from Placenta Tissue Samples of Women in Groups III, IV, and V

To confirm the correlations revealed for the expression levels of miR-17-5p, miR-21-5p, miR-25-3p, miR-92a-3p, and miR-320a-3p with the severity of AIP and to prove the placental origin of these miRNAs, deep sequencing of small noncoding RNAs from the placenta in the area of pathological invasion (P-area) and outside this area (N-area) was performed. Placental samples (N and P) from eight women from groups III, IV, and V were taken for sequencing, and the read numbers of miRNAs in these samples processed by the DESeq2 package [34] are presented in Table S1 for all identified miRNAs, and in Table 7 and Table 8 for miR-17-5p, miR-21-5p, miR-25-3p, miR-92a-3p, and miR-320a-3p. The fold changes in the miRNA expression level in groups IV and V relative to group III and the statistical significance of these differences were calculated. It follows from Table 7 that with increasing AIP severity, the expression levels of miR-17-5p, miR-21-5p, miR-25-3p and miR-92a-3p, miR-320a-3p in the P-areas significantly increased. Increases in the expression levels of these miRNAs in groups IV and V relative to group III were also observed in the N-areas, but these changes were comparable, and in such cases, statistically significant increases in the expression levels in both groups were observed only for miR-25-3p and miR-92a-3p (Table 8).

### 3.5. Quantitative Analysis of Potential Targets of miRNAs—Participants in the EMT

In order to analyze the expression levels of potential targets of miR-17-5p, miR-21-5p, miR-25-3p, miR-92a-3p, and miR-320a-3p, the contents of the soluble form of E-cadherin and the alpha subunit of the secretory form of clusterin were quantified by Western blotting in the same peripheral blood plasma samples in which the expression levels of miRNAs regulating them were analyzed.

#### 3.5.1. E-Cadherin Quantitation in the Peripheral Blood Plasma of Pregnant Women

The E-cadherin monoclonal antibody used in the present study was raised against the extracellular domain of E-cadherin, shedding into the blood plasma as a result of proteolysis of the full-length form by any of a number of proteases including MMP3, MMP7, MMP9, plasmin, kallikrein, MT-1-MMP, ADAM10, caspases, and calpain, leading to the formation of E-cadherin fragments with different molecular weights [40,41,42,43,44].

**Figure 5:**
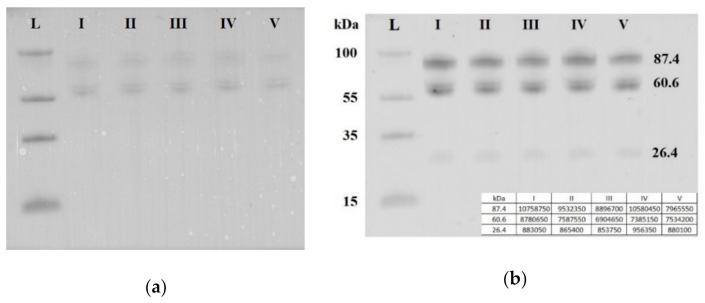
Western blot analysis of soluble E-cadherin in peripheral blood plasma pooled samples from groups I–V. Blot was stained with Ponceau S dye (**a**) and with antibody against the extracellular domain of E-cadherin (**b**). Мolecular weights (kDa) of the protein ladder and fragments of soluble E-cadherin are indicated on the blot.

Seven blood plasma samples from each of the groups I–V were pooled and analyzed by Western blotting. The same quantity loading of total protein into the wells of the PAAG/SDS and the efficiency of semi-dry transfer of proteins from the gel to the PVDF membrane were controlled by staining the membrane with Ponceau S dye (Figure 5a). It follows from Figure 5b that three soluble fragments of E-cadherin with molecular weights of 87.4, 60.6 and 26.4 kDa were revealed in all studied sample groups. Quantitative analysis of chemiluminescence showed no obvious differences between the analyzed groups in the content of each of the three fragments of soluble E-cadherin (the data are summarized in the table presented in the inset of the Figure 5b). Therefore, a detailed analysis of the content of soluble E-cadherin individually in each sample of the analyzed groups I-V was not carried out.

#### 3.5.2. Quantitation of the Clusterin Secretory form in the Peripheral Blood Plasma of Pregnant Women

There are two isoforms of clusterin synthesized in the cell as a result of alternative splicing: the secretory and nuclear forms. The nuclear form of clusterin is a low-glycosylated protein and is translocated from the cytoplasm to the nucleus. The secretory form of clusterin is a glycosylated protein consisting of α and β chains linked by five disulfide bonds.

**Figure 6:**
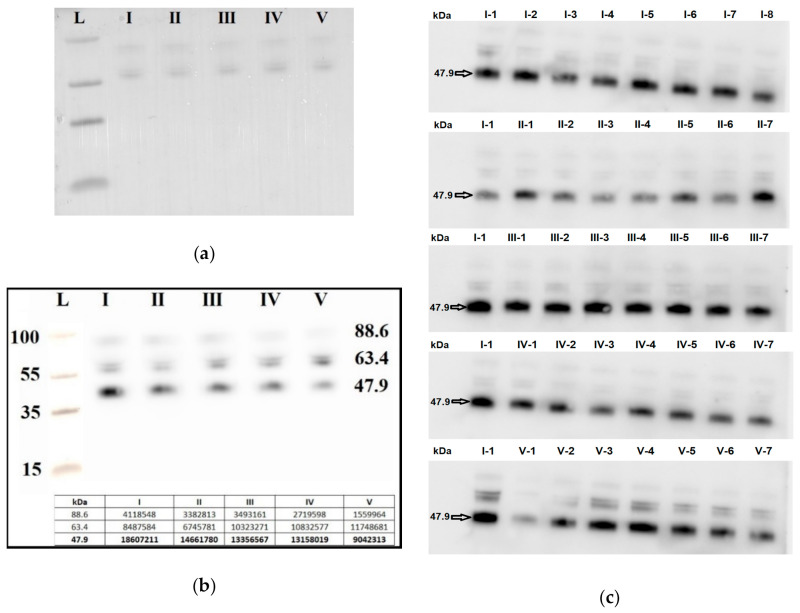
Western blot analysis of the α-subunit of the clusterin secretory form in peripheral blood plasma samples from groups I–V. Blot with pooled samples from groups I-V was stained with Ponceau S dye (**a**) and with antibody against the α-subunit of the clusterin secretory form (**b**). Blots with individual samples from each group I–V were stained with antibody against the α-subunit of the clusterin secretory form (**c**), wherein arabic numerals, written with a hyphen next to the group number, indicate the sample numbers. Мolecular weights (kDa) of the protein ladder and clusterin are indicated on the blots.

Seven blood plasma samples from each of the groups I–V were pooled and analyzed by Western blotting. The same quantity loading of total protein into the wells of the PAAG/SDS and the efficiency of semi-dry transfer of proteins from the gel to the PVDF membrane were controlled by staining the membrane with Ponceau S dye (Figure 6a). It follows from Figure 6b that the level of the α-subunit of the clusterin secretory form with molecular weight of 47.9 kDa was decreased in all samples with AIP (groups III–V) relative to group I (chemiluminescence data are presented in the inset of Figure 6b). Therefore, a detailed analysis of the content of the α-subunit of the clusterin secretory form individually in each sample of the analyzed groups I–V was carried out.

Seven blood plasma samples from each of the groups I–V were taken into analysis. To take into account the efficiency of protein transfer from PAAG/SDS to the PVDF membrane, the binding of primary and secondary antibodies, exposure in the analysis of chemiluminescence, the same sample (number 1 from group I, I-1) was applied to each PAAG/SDS to normalize all studied samples (Figure 6c).

**Figure 7:**
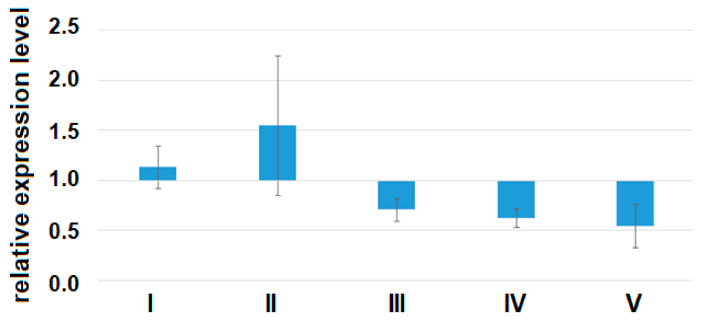
The expression level of the α-subunit of the clusterin secretory form in the peripheral blood plasma from women of groups I–V analyzed by Western blotting. Data are presented as Me ± standard deviation (stdev).

A quantitative assessment of the alpha subunit of the secretory form of clusterin (47.9 kDa) in the peripheral blood plasma of pregnant women revealed a statistically significant decrease in the level of clusterin expression in all analyzed groups of women with AIP (III–V) relative to group I or group II (Figure 7, Table 9).

Using the Spearman rank correlation method, significant positive correlations of the content of clusterin in the peripheral blood plasma with the miRNA ΔCt values were found, namely for miR-21-5p (r = 0.74, p < 0.001), miR-25-3p (r = 0.54, p = 0.001), miR-92a-3p (r = 0.69, p < 0.001), miR-320a-3p (r = 0.64, p < 0.001), and miR-17-5p (r = 0.53, p = 0.001), which indicates that there were inverse correlations between the expression levels of these miRNAs and the concentration of clusterin. At the same time, an inverse correlation was found between the expression level of the α-subunit of clusterin and the severity of AIP (r = −0.79, p < 0.001). Inverse correlations between the expression level of miR-25-3p, miR-92a-3p, miR-320a-3p, miR-17-5p, and clusterin may not be a consequence of the direct interaction of these molecules, while the existence of experimentally validated interactions between hsa-miR-21-5p, hsa-miR-17-5p, and clusterin according to the miRTargetLink Human database have been already proven. This fact is not an obstacle for AIP diagnostics, because for this purpose, it is important to reveal the differential expression level of miR-25-3p, miR-92a-3p, miR-320a-3p, miR-17-5p, and clusterin, rather than the physical interaction of molecules.

### 3.6. Development of Logistic Regression Models for Each form of AIP

**Figure 8:**
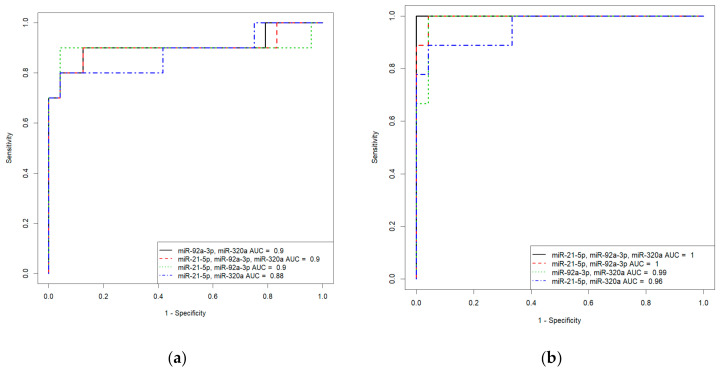

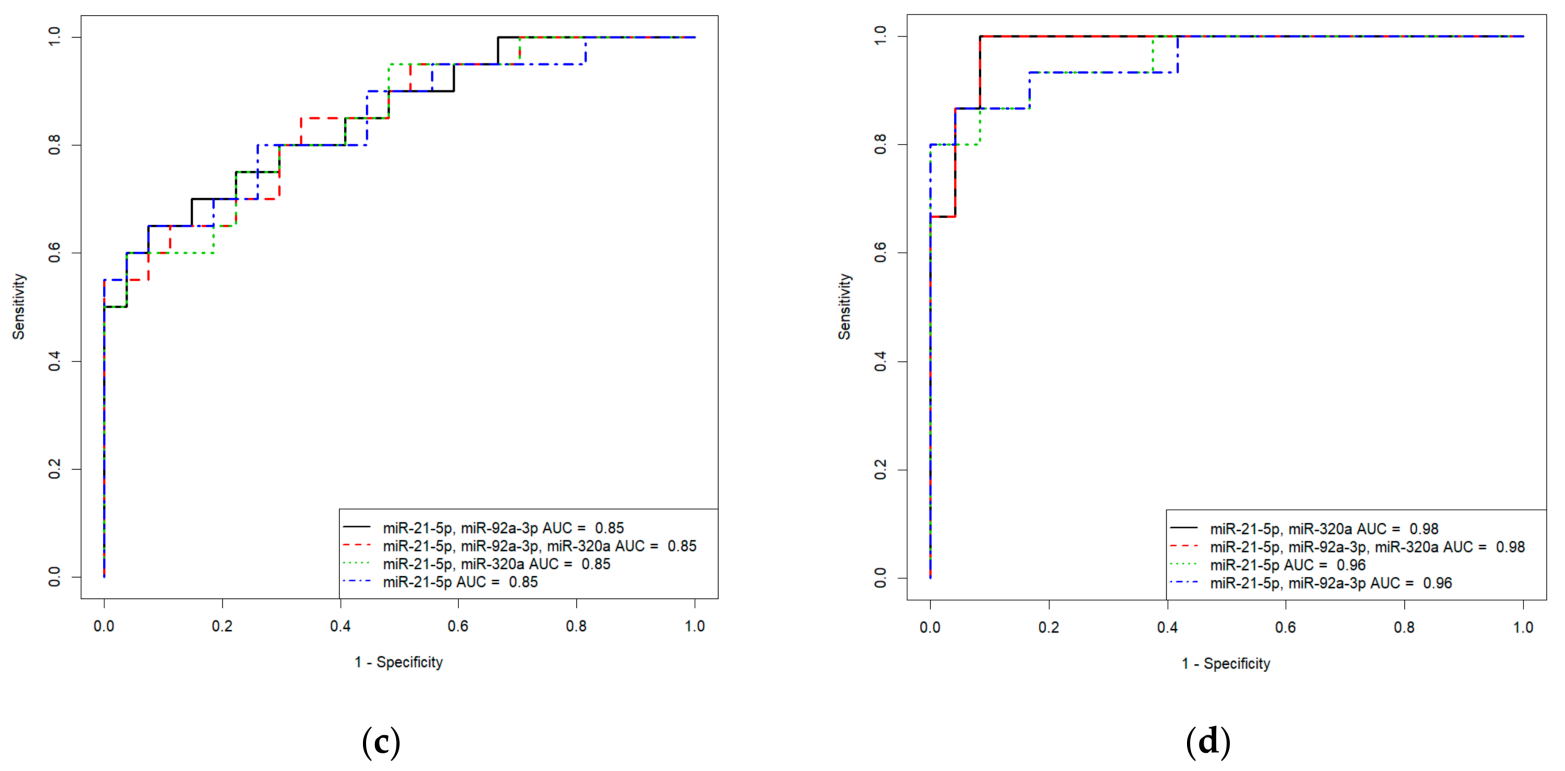
Receiver operating characteristic (ROC) curves of the logistic regression models in case of entire cohort of patients (**a**,**c**) and optimized cohort of patients (**b**,**d**) for placenta accreta (**a**,**b**) and placenta increta (**c**,**d**).

Based on the values of clusterin content and the expression levels of its potential regulators, miR-21-5p, miR-92a-3p, and miR-320a-3p, in the peripheral blood plasma, due to their highly significant correlations with the severity of AIP, logistic regression models were developed for calculating the probability of the presence of placenta accrete, increta, or percreta in a pregnant woman at 32–34 weeks of gestation. Based on the expression levels of miR-21-5p, miR-92a-3p, and miR-320a-3p, logistic regression models were developed for the entire cohort of patients, including pregnant women with unconfirmed MRI/ultrasound diagnoses of AIP during cesarean section and/or histological analysis (Table S2, Sheet 1: patients without AIP, *n* = 27; patients with placenta accreta, *n* = 10; patients with placenta increta, *n* = 20; patients with placenta percreta, *n* = 7), and for an optimized cohort of patients (Table S2, Sheet 2: patients without AIP, *n* = 24; patients with placenta accreta, *n* = 9; patients with placenta increta, *n* = 14; patients with placenta percreta, *n* = 7 ). It seemed interesting to find out whether the classification of samples by the presence or absence of AIP, based only on MRI/ultrasound data, affected the parameters of the developed logistic regression models. We found that the diagnostic accuracy of expression profiling of the combination of miR-21-5p, miR-92a-3p, and miR-320a-3p for the identification of placenta accreta was higher in the case of optimized cohort of patients (AUC = 1, *p* < 0.001; accuracy—100 %, sensitivity—100%, specificity—100%, true positive rate—1; false positive rate—0) compared to that of the case of entire cohort of patients (AUC = 0.9, *p* < 0.001; accuracy—88.2 %, sensitivity—90%, specificity—95.8%, true positive rate—0.9; false positive rate—0.125). Similarly, the diagnostic accuracy of expression profiling of the combination of miR-21-5p, miR-92a-3p, and miR-320a-3p for the identification of placenta increta was significantly higher in case of optimized cohort of patients (AUC = 0.981, *p* < 0.001; accuracy—94.9 %, sensitivity—100%, specificity—91.6%, true positive rate—1; false positive rate—0.0833) compared to that in case of entire cohort of patients (AUC = 0.848, *p* < 0.001; accuracy—80.9 %, sensitivity—75%, specificity—92.5%, true positive rate—0.55; false positive rate—0). Receiver operating characteristic (ROC) curves of the developed logistic regression models in cases of entire and optimized cohorts of patients are presented in Figure 8. The diagnostic accuracy of expression profiling of each of miR-21-5p, miR-92a-3p, miR-320a-3p for the identification of placenta percreta was the same for the two cohorts of patients (Table S1, Sheet 1 and Sheet 2: AUC = 1, *p* < 0.001; accuracy—100 %, sensitivity—100%, specificity—100%, true positive rate—1; false positive rate—0).

The α-subunit of the clusterin secretory form expression profile was also able to differentiate between patients with AIP and patients without AIP. In particular, the following parameters of the developed models were obtained: AUC = 1 (*p* < 0.001), accuracy—100 %, sensitivity—100%, specificity—100%, true positive rate—1, false positive rate—0, in the cases of placenta accreta and placenta increta; AUC = 0.987 (*p* < 0.001), accuracy—94.7%, sensitivity—100%, specificity—100%, true positive rate—1, false positive rate—0.076, in the case of placenta percreta (Table S1, Sheet 3).

The general Formula (1) of these models was as follows:

(1) $$e=\frac{1}{1+e^{-i-k1*x1-k2*x2-\ldots }},$$

where i is the intercept term; k1 and k2 are coefficients for clusterin or each of the miRNAs; and x1, x2, … are the relative expression levels of clusterin or miRNA ΔCt values.

The parameters of the developed models for optimized cohort of patients that reflect the contributions of the expression level of clusterin or miRNAs to diagnose the different forms of AIP are presented in Table 10.

It follows from Table 10 that the different forms of AIP (placenta accreta, increta, percreta) in a pregnant woman at 31–34 weeks of gestation can be diagnosed by the content of the α-subunit of the clusterin secretory form or by the expression level of miR-21-5p, miR-320a-3p, and miR-92a-3p in various combinations in the peripheral blood plasma with a sensitivity of 88.8–100% and a specificity of 91.6–100% at the corresponding cutoff level indicated in this Table 10. This type of analysis significantly increases the diagnostic accuracy of conventional ultrasound and MRI methods, the sensitivity and specificity of which vary in the range of 33–93% Se and 71–100% Sp.

## 4. Discussion

Research groups are trying to determine the underlying cause of AIP: (i) morphological changes in the decidua due to endometritis and/or antecedent curettage; (ii) incompetent uterine scar after a cesarean section; or (iii) the properties of placental tissue characterized by increased proliferative and reduced apoptotic activity of extravillous trophoblast cells along with abnormally aggressive EMT of these type of cells which does not stop by the end of the first trimester of pregnancy but occurs throughout the entire pregnancy [49,50]. Most likely, in our opinion, chronic inflammation in the uterus leads to pathological invasiveness of the placenta after embryo implantation. This assumption is supported by data showing that in the site of chronic inflammation due to damage, macrophages and activated resident fibroblasts secrete growth factors (TGF-β, PDGF, EGF, FGF-2), chemokines, and matrix metalloproteinases (MMP-2, -3, -9), the result of which is the activation of signaling pathways (receptor tyrosine kinase pathway, Wnt-, Notch-, TGFβ-, Hedgehog, TNFα-pathway) [17] leading to the synthesis of epithelial repressors (Snail, Slug, Smuc, Twist, E12/E47, dEF1, ZEB1, ZEB2), downregulation of epithelial genes (E-cadherin, EpCAM, Claudins, Cytokeratins), and activation of mesenchymal genes (N-cadherin, Vimentin, Fibronectin, MMPs), which causes a highly invasive cell phenotype [16,51]. In this regard, it seems reasonable to diagnose AIP using a noninvasive method of quantitatively evaluating potential participants in the EMT in trophoblast cells, in particular, clusterin and E-cadherin as well as potential regulators of their expression—hsa-miR-21-5p, hsa-miR-25-3p, hsa-miR-92a-3p, hsa-miR-320a-3p, and hsa-miR-17-5p—in the peripheral blood plasma of pregnant women.

Mature human E-cadherin is expressed as a glycoprotein (120 kDa) which consists of five extracellular domains, a linker region, a single transmembrane domain, and an intracellular domain. E-cadherin plays an important role in cell–cell contact formation by “homophilic adhesion” through the extracellular domains. E-cadherin can undergo intracellular and extracellular proteolytic cleavage, providing an additional mechanism to transcription repression regulation to reduce cell surface expression [52,53]. The catalytic activity of the E-cadherin-cleaving proteases triggers the extracellular release of soluble E-cadherin fragments of different molecular weights depending on the cleavage site: for MMPs, it is between aa residues 581 and 583 [54] and/or between aa residues 700 and 701 [55]; for γ-secretase, it is between aa residues 731 and 732 [55]; for caspase 3, it is between aa residues 750 and 751 [56]; and for PMN–elastase (leukocyte–elastase), it is between Val-393 and Gly-394 [57]. It is obvious that the expression levels of full-length E-cadherin and its soluble forms shed into the blood are determined by the balance between the biosynthesis and proteolytic cleavage activity of these proteases and may be crucial determinants in the adhesion and migration of cells. Extracellular release of a soluble E-cadherin fragment of about 80 kDa from the cell surface is accompanied by the simultaneous delivery of free β-catenin into the cell cytosol, which then translocates into the cell nucleus where it contributes to the modulation of gene expression [58]. In the present study, in the blood plasma of pregnant women, we found three soluble N-terminal E-cadherin fragments of 87.4, 60.6 and 26.4 kDa, and the ratio of these forms did not change in the blood plasma of pregnant women with any form of AIP in comparison with healthy pregnant women. These data are in good agreement with the absence of significant difference in circulatory soluble E-cadherin levels detected in the serum of women with normal placentation, invasive placentation, and previa alone [19], suggesting that an invasive phenotype of extravillous trophoblasts is not systemically regulated and could be the result of local processing of E-cadherin. It is known that E-cadherin has four utilized N-glycosylation sites, and removal of these sites decreases the functional E-cadherin level at the cell–cell border [59]. In addition, it has been shown that a decrease in the expression level of the membrane-bound form of E-cadherin is not sufficient for the effective regulation of EMT [60,61].

The role of another protein analyzed in this work, clusterin, in the regulation of EMT is discussed mainly in the context of carcinogenesis. Clusterin (apolipoprotein J) is involved in various physiological cellular processes; its function is determined by the structure of the molecule itself [62]. There are two isoforms of clusterin (secretory and nuclear), the transcription of which occurs from different promoter regions of the gene, forming mRNAs that differ in the sequence of the first exon and in the location of the translation initiation site. The secretory form of clusterin is a 76–80 kDa glycosylated protein consisting of α and β chains linked by five disulfide bonds. The nuclear form (55 kDa) of clusterin is a low-glycosylated protein that, after synthesis in the cytoplasm, is translocated into the nucleus. The nuclear form of clusterin performs proapoptotic functions, while the secretory form of clusterin is responsible for the survival of the cell and its proliferation. The prevalence of one form or another in the cell is finely regulated, and it remains a mystery as to what determines their synthesis. For example, it has been shown that, during oncogenic transformation, tumor cell survival is associated with increased expression of the secretory form of clusterin and significant decrease in the level of expression of the nuclear form of clusterin [63].

The expression level of clusterin is regulated at the transcriptional and post-transcriptional levels. The regulators of clusterin transcription are the p53 apoptotic cascade activator [64] and the B-MYB transcription factor, which are involved in the regulation of cell survival, proliferation, and differentiation [65]. In turn, clusterin regulates the activity of the transcription factor NF-κB, which plays important roles in cell viability, cell motility, proliferation, transformation, and inflammation [66].

The specificity of clusterin expression for syncytiotrophoblast cells and chorionic villus endothelium has been proven [30], and its increased expression was also found in preeclampsia both in the placenta [30] and in peripheral blood plasma [31], the key pathogenetic mechanism of which is dysregulation of the phenotypic transformation of trophoblast cells and a reduction in their invasive ability. Until now, there were no data on changes in the expression and role of this protein in the development of AIP. In the present study, for the first time, a significant decrease in the expression level of the secretory form of clusterin was revealed in pregnant women with placenta accreta, increta, and percreta, compared to pregnant women with a scar on the uterus and/or placenta previa.

The expression level of clusterin, like any other protein, can be regulated at the post-transcriptional level by the activity of small noncoding RNAs. In this work, a significant inverse correlation was revealed between the expression level of clusterin and its potential regulators: hsa-miR-21-5p, hsa-miR-25-3p, hsa-miR-92a-3p, hsa-miR-320a-3p, and hsa-miR-17-5p. In a review article by Minal Garg [16], the involvement of miR-21-5p, miR-25-3p, miR-92a-3p, miR-320a-3p, and miR-17-5p in the induction of EMT by either suppressing or activating several signaling genes is described in detail. Moreover, downregulation of miR-21-5p has been found to inhibit cancer cell proliferation and induce apoptosis by suppressing the ERK and PI3K/Akt signaling pathways, elevating full-length E-cadherin expression, reducing the expression of the mesenchymal marker vimentin as well as MMP-2 and MMP-9, and acting as an extracellular inducer of MMPs [67]. Increased expression of miR-92a-3p contributes to the EMT and metastasis in colorectal cancer through activation of the Wnt/β-catenin pathway and inhibition of mitochondrial apoptosis [68] as well as in hepatocellular carcinoma and malignant retinoblastoma through the PTEN/Akt pathway [69,70]. Blood plasma exosomes from breast cancer patients and healthy donors were found to differ in the overexpression of miR-25-3p and miR-92a-3p, and this was related to the stimulation of the EMT of nonmalignant breast cells, significant increases in the number of motile and proliferating cells, as well as the formation of capillary-like structures [71].

In addition, changes in the expression levels of these molecules in diseases associated with changes in cell adhesion and migration have been proven. Specifically, there are decreases in the expression levels of miR-92a-3p, miR-17-5p [72], miR-21-5p [73], and miR-320a-3p [74] in the placenta during preeclampsia. Thus, in AIP cases characterized by increased invasion of extravillous trophoblast cells, we identified expression profiles of hsa-miR-21-5p, hsa-miR-25-3p, hsa-miR-92a-3p, hsa-miR-320a-3p, and hsa-miR-17-5p and their target gene clusterin that were directly opposite to those seen in preeclampsia, the pathogenesis of which is based on reduced invasion of extravillous trophoblast cells. This underlines the importance of using these molecules for diagnostic purposes. Related to this connection, we constructed logistic regression models to diagnose AIP forms of varying levels of severity (placenta аccreta, increta, percreta) at 32–34 weeks with high specificity and sensitivity based on a quantitative evaluation of the secretory form of clusterin and its regulatory miRNAs—hsa-miR-21-5p, hsa-miR-92a-3p, and hsa-miR-320a-3p—in the peripheral blood plasma of pregnant women. The data obtained in thе present work significantly contribute to improvements in the accuracy of AIP diagnosis by instrumental methods [75] and the analysis of placental and fetal hormones (pregnancy-associated plasma protein A, human chorionic gonadotropin, alpha-fetoprotein) by cell-free placental mRNAs [49] as well as by recently discovered plasma marker miRNAs, in particular, miR-139-3p, miR-196a-5p, miR-518a-3p, and miR-671-3p, which are downregulated in placenta increta/percreta and have the potential to be used for future noninvasive prenatal placenta accreta spectrum screening [76].

## 5. Conclusions

1. The expression levels of hsa-miR-17-5p, hsa-miR-21-5p, hsa-miR-25-3p, hsa-miR-92a-3p, and hsa-miR-320a-3p were significantly increased in the peripheral blood plasma of pregnant women with AIP compared to their concentrations in the blood plasma of pregnant women with placenta previa without AIP. This correlated significantly with the severity of AIP. With an increase in the severity of AIP, the expression levels of these miRNAs increased in the placenta in the area of pathological invasion.

2. The content and ratio of soluble E-cadherin fragments in the blood plasma of pregnant women with any form of AIP did not differ from those in the blood plasma of healthy pregnant women.

3. The level of the secretory form of clusterin was significantly reduced in the peripheral blood plasma of pregnant women with AIP compared to that in the blood plasma of pregnant women without AIP. This significantly inversely correlated with the expression levels of hsa-miR-17-5p, hsa-miR-21-5p, hsa-miR-25-3p, hsa-miR-92a-3p, and hsa-miR-320a-3p and with the severity of AIP.

4. The probability of different forms of AIP (placenta accreta, increta, percreta) occurring in a pregnant woman at 31–34 weeks of gestation could be evaluated by the content of the α-subunit of the clusterin secretory form or by the expression levels of hsa-miR-21-5p, hsa-miR-320a-3p, and hsa-miR-92a-3p in various combinations in peripheral blood plasma from pregnant women with a sensitivity level of 88.8–100% and a specificity level of 91.6–100%, which increases the diagnostic accuracy of conventional ultrasound and MRI methods.

5. It is reasonable to quantify the secretory form of clusterin and the miRNAs that regulate its expression level in the first trimester of pregnancy with the aim of assessing their predictive value in determining AIP development.

## Figures and Tables

**Table 1:** Clinical characteristics of the pregnant women.

Group of Pregnant Women ^1^	AIP	Scar on the Uterus	Placenta Previa	Age ^2^	Number of Pregnancies in History ^2^	Number of Previous Cesarean Sections ^2^
I (*n* = 10)	abs	yes	no	36.5 (33; 39)	3 (3; 3.8)	1 (1; 1.8)
IIa (*n* = 9)	abs	no	yes	36 (36; 39)	3 (1; 3)	0 (0; 0) **
IIb (*n* = 8)	abs	yes	yes	37.5 (34; 38.3)	3 (2.8; 6.3)	2 (1; 2.3)
III (*n* = 10)	accreta	yes	yes	34 (33; 35.8)	4 (3.3; 5)	2 (2; 3) *
IV (*n* = 20)	increta	yes	yes	33 (29.5; 36.3)	4 (3; 5)	2 (1; 2)
V (*n* = 7)	percreta	yes	yes	33 (32; 33)	4 (2.5; 4)	2 (1.5; 2)

^1^ Roman numerals indicate the group number; the size of the group is indicated in brackets. ^2^ Data are presented as the median (Me) and quartiles Q1 and Q3 in the format: Me (Q1; Q3). * The value of statistical significance of differences when compared to group I is less than 0.05. ** The value of statistical significance of differences when compared to group I is less than 0.0001.

**Table 2:** miRNA parameters.

miRNA	miRNA Gene Target	miRNA Accession Number (miRBase)	Nucleotide Sequence of Sense Primer for PCR, 5′-3′	PCR Primers Annealing Temperature, ℃
miR-320a-3p	*CDH*, *CLU*	MIMAT0000510	aaaagctgggttgagagggcga	59.5
miR-17-5p	*CDH*, *CLU*	MIMAT0000070	caaagtgcttacagtgcaggtag	55
miR-21-5p	*CDH*, *CLU*	MIMAT0000076	tagcttatcagactgatgttga	54.6
miR-1323	*CDH*, *CLU*	MIMAT0005795	tcaaaactgaggggcattttct	51
miR-25-3p	*CDH*, *CLU*	MIMAT0000081	cattgcacttgtctcggtctga	56
miR-138-5p	*CDH*, *CLU*	MIMAT0000430	agctggtgttgtgaatcaggccg	54.6
miR-34a-5p	*CDH*, *CLU*	MIMAT0000255	tggcagtgtcttagctggttgt	51.3
miR-92a-3p	*CDH*, *CLU*	MIMAT0000092	tattgcacttgtcccggcctgt	60
miR-30a-5p	*CLU*	MIMAT0000087	tgtaaacatcctcgactggaag	54.6
miR-30c-5p	*CLU*	MIMAT0000244	tgtaaacatcctacactctcagc	49.1
miR-371a-5p	*CDH*	MIMAT0004687	actcaaactgtgggggcact	54.6
miR-506-3p	*CDH*	MIMAT0002878	taaggcacccttctgagtaga	51.3
miR-382-5p	endogenous control for PCR	MIMAT0000737	gaagttgttcgtggtggattcg	49.1

**Table 3:** Pairwise comparison of groups II–V to group I by the miRNA ΔCt value for an optimized cohort of patients.

miRNA	Group	Ме	Q1	Q3	Mann–Whitney Test, *p*-Value	Bonferroni Adjustments for 20 Tests (5 miRNAs & 4 Comparisons), *p*-Value
miR-17-5p	I	10.95	6.23	11.04		
miR-17-5p	IIa + IIb	11.07	10.87	11.19	0.055163	1
miR-17-5p	III	8	6.51	10.85	0.135911	1
miR-17-5p	IV	7.02	5.44	10.75	0.040858	0.81716
miR-17-5p	V	9.67	5.24	9.75	0.054895	1
miR-21-5p	I	10.89	7.23	11.03		
miR-21-5p	IIa + IIb	8.55	7.4	11.06	0.725852	1
miR-21-5p	III	5.2	4.42	5.93	<0.0001	0.0198
miR-21-5p	IV	5.39	4.46	5.89	<0.0001	<0.0001
miR-21-5p	V	0.67	0.4	0.99	0.000175	0.0035
miR-25-3p	I	7.28	6.33	8.53		
miR-25-3p	IIa + IIb	8.77	7.11	10.87	0.317547	1
miR-25-3p	III	5.94	5.06	6.35	0.077005	1
miR-25-3p	IV	6.34	4.71	9.45	0.289503	1
miR-25-3p	V	1.65	0.91	2.09	0.000175	0.0035
miR-320a-3p	I	4	3.39	4.49		
miR-320a-3p	IIa + IIb	3.91	3.66	4.66	0.815253	1
miR-320a-3p	III	2.75	2.29	2.94	0.000494	0.00988
miR-320a-3p	IV	2.84	2.6	3.22	<0.0001	0.00092
miR-320a-3p	V	1.01	0.92	1.69	0.000175	0.0035
miR-92a-3p	I	3.74	3.71	4.04		
miR-92a-3p	IIa + IIb	4.17	3.75	4.4	0.215228	1
miR-92a-3p	III	2.82	2.61	3	0.000165	0.0033
miR-92a-3p	IV	2.98	2.62	3.64	0.025147	0.50294
miR-92a-3p	V	−0.01	−0.58	0.48	0.000175	0.0035

Data are presented as the median (Me) of the ΔCt values and quartiles Q1 and Q3 and the statistical significance of the differences p while applying Mann–Whitney test with Bonferroni correction for multiple testing.

**Table 4:** Holm–Bonferroni test for pairwise comparison of groups II–V to group I by the miRNA ΔCt value from Table 3.

**miRNA**	**I vs. II, Mann–Whitney Test, *p*-Value**	**Order**	**Holm–Bonferroni Test, *p*-Value**	**Significance ^1^**
miR-17-5p	0.05516	1	0.01000	0
miR-92a-3p	0.21523	2	0.01250	0
miR-25-3p	0.31755	3	0.01667	0
miR-21-5p	0.72585	4	0.02500	0
miR-320a-3p	0.81525	5	0.05000	0
**miRNA**	**I vs. III, Mann–Whitney Test, *p*-Value**	**Order**	**Holm–Bonferroni Test, *p*-Value**	**Significance ^1^**
miR-21-5p	0.0001	1	0.01000	1
miR-92a-3p	0.0002	2	0.01250	1
miR-320a-3p	0.0005	3	0.01667	1
miR-25-3p	0.0770	4	0.02500	0
miR-17-5p	0.1359	5	0.05000	0
**miRNA**	**I vs. IV, Mann–Whitney Test, *p*-Value**	**Order**	**Holm–Bonferroni Test, *p*-Value**	**Significance ^1^**
miR-21-5p	0.00010	1	0.01000	1
miR-320a-3p	0.00010	2	0.01250	1
miR-92a-3p	0.02515	3	0.01667	0
miR-17-5p	0.04086	4	0.02500	0
miR-25-3p	0.28950	5	0.05000	0
**miRNA**	**I vs. V, Mann–Whitney Test, *p*-Value**	**Order**	**Holm–Bonferroni Test, *p*-Value**	**Significance ^1^**
miR-21-5p	0.00018	1	0.01000	1
miR-92a-3p	0.00018	2	0.01250	1
miR-320a-3p	0.00018	3	0.01667	1
miR-25-3p	0.00018	4	0.02500	1
miR-17-5p	0.05490	5	0.05000	0

^1^ “1” means statistically significant difference, “0” means insignificant difference.

**Table 5:** Pairwise comparison of groups III–V to group II by miRNA ΔCt values for the optimized cohort of patients.

miRNA	Group	Ме	Q1	Q3	Mann–Whitney Test, *p*-Value	Bonferroni Adjustments for 15 Tests (5 miRNAs & 3 Comparisons)
miR-17-5p	IIa + IIb	11.07	10.87	11.19		
miR-17-5p	III	8	6.51	10.85	0.00167	0.02499
miR-17-5p	IV	7.02	5.44	10.75	0.00014	0.0021
miR-17-5p	V	9.67	5.24	9.75	0.00289	0.043275
miR-21-5p	IIa + IIb	8.55	7.4	11.06		
miR-21-5p	III	5.2	4.42	5.93	0.00015	0.00222
miR-21-5p	IV	5.39	4.46	5.89	<0.0001	<0.0001
miR-21-5p	V	0.67	0.4	0.99	<0.0001	<0.0001
miR-25-3p	IIa + IIb	8.77	7.11	10.87		
miR-25-3p	III	5.94	5.06	6.35	0.00826	0.12383
miR-25-3p	IV	6.34	4.71	9.45	0.08920	1
miR-25-3p	V	1.65	0.91	2.09	<0.0001	0.00117
miR-320a-3p	IIa + IIb	3.91	3.66	4.66		
miR-320a-3p	III	2.75	2.29	2.94	0.00029	0.00441
miR-320a-3p	IV	2.84	2.6	3.22	<0.0001	0.00396
miR-320a-3p	V	1.01	0.92	1.69	<0.0001	<0.0001
miR-92a-3p	IIa + IIb	4.17	10.87	11.19		
miR-92a-3p	III	2.82	6.51	10.85	0.00015	0.00222
miR-92a-3p	IV	11.07	5.44	10.75	0.00316	0.04733
miR-92a-3p	V	8	5.24	9.75	<0.0001	0.00093

Data are presented as the median (Me) of the ΔCt values and quartiles Q1 and Q3 and the statistical significance of the differences p while applying Mann–Whitney test with Bonferroni correction for multiple testing.

**Table 6:** Holm–Bonferroni test for pairwise comparison of groups II–V to group I by the miRNA ΔCt value from Table 5.

**miRNA**	**II vs. III, Mann–Whitney Test, *p*-Value**	**Order**	**Holm–Bonferroni Test, *p*-Value**	**Significance**
miR-21-5p	0.00015	1	0.01	1
miR-92a-3p	0.00015	2	0.0125	1
miR-320a-3p	0.00029	3	0.01667	1
miR-25-3p	0.00826	4	0.025	1
miR-17-5p	0.00167	5	0.05	1
**miRNA**	**II vs. IV, Mann–Whitney Test, *p*-Value**	**Order**	**Holm–Bonferroni Test, *p*-Value**	**Significance**
miR-21-5p	<0.0001	1	0.01	1
miR-320a-3p	<0.0001	2	0.0125	1
miR-17-5p	0.00014	3	0.01667	1
miR-92a-3p	0.00316	4	0.025	1
miR-25-3p	0.08920	5	0.05	0
**miRNA**	**II vs. V, Mann–Whitney Test, *p*-Value**	**Order**	**Holm–Bonferroni test, *p*-Value**	**Significance**
miR-21-5p	<0.0001	1	0.01	1
miR-320a-3p	<0.0001	2	0.0125	1
miR-92a-3p	<0.0001	3	0.01667	1
miR-25-3p	<0.0001	4	0.025	1
miR-17-5p	0.00289	5	0.05	1

“1” means a statistically significant difference, “0” means an insignificant difference.

**Table 7:** Deep sequencing data on the miRNA expression level in the placenta in the area of pathological invasion (P-area).

miRNA ^1^	III_P1	III_P2	III_P3	IV_P4	IV_P5	IV_P8	V_P6	V_P7	FC (IV/III)	FC (V/III)	*p* (IV vs. III)	*p* (V vs. III)	
miR-17-5p	255	255	328	268	1000	1287	1734	1567	3.9	6.5	0.026	<0.001	
miR-21-5p	18,635	11,690	8478	6522	12,449	16,980	23,703	25,653	1.1	2.1	0.851	0.030	
miR-25-3p	2988	1954	1830	1280	47,991	82,556	75,469	108,393	24.6	47.0	<0.001	<0.001	
miR-92a-3p	19,470	19,215	34,320	23,702	453,638	590,837	857,646	911,579	23.3	45.4	<0.001	<0.001	
miR-320a-3p	10,183	11,134	11,309	14,033	19,978	21,489	39,849	33,092	1.8	3.3	0.023	<0.001	

^1^ III, IV, and V are the group numbers; P1–P8 are the numbers of placenta samples from the P-area; FC is the fold change in the expression level as the ratio of the medians of the miRNA read numbers in the compared groups; *p*—statistical significance of differences.

**Table 8:** Deep sequencing data on miRNA expression level in the placenta in the area outside of pathological invasion (N-area).

miRNA ^1^	III_N1	III_N2	III_N3	IV_N4	IV_N5	IV_N8	V_N6	V_N7	FC (IV/III)	FC (V/III)	*p* (IV vs. III)	*p* (V vs. III)
miR-17-5p	301	218	212	208	741	1122	767	389	3.4	2.7	0.104	0.048
miR-21-5p	8290	6505	6265	11,066	17,856	15,617	26,978	7142	2.4	2.6	0.158	0.064
miR-25-3p	2451	1716	1222	1211	40,054	84,350	49,601	2940	23.3	15.3	<0.001	<0.001
miR-92a-3p	15,612	18,053	27,168	22,394	288,269	722,332	647,513	14,940	16.0	18.3	<0.001	0.002
miR-320a-3p	6485	8611	10,781	15,287	30,893	22,918	29,831	5513	2.7	2.1	0.075	0.191

^1^ III, IV, and V are the group numbers; P1–P8 are the numbers of placental samples from the N-area; FC is the fold change in the expression level as the ratio of the medians of the miRNA read numbers in the compared groups; *p*—statistical significance of differences.

**Table 9:** Pairwise comparison of groups III–V to group I or group II by the expression level of the α-subunit of the clusterin secretory form in the peripheral blood plasma.

	I	II	III	IV	V
Me	1.13727	1.55384	0.70564	0.62291	0.54514
stdev	0.21091	0.69933	0.11278	0.09199	0.21387
*p* *		0.06561	<0.001	<0.001	<0.001
*p* **			0.00405	0.00222	0.00167

* statistical significance of differences between groups II–V with group I. ** statistical significance of differences between groups III–V with group II.

**Table 10:** Parameters of the logistic regression models.

Biomarker	AUC	Sp	Se	Cutoff	i	К
Placenta accreta						
“Clusterin”	1	1	1	0.5	1832.89	−2232.65
“miR-21-5p + miR-92a-3p + miR-320a-3р”	1	1	1	0.5	4526.11	−141.53 −868.31 −244.95
“miR-21-5p + miR-92a-3p”	0.995	1	1	0.262	64.43	−3.32 −13.08
“miR-92a-3p + miR-320a-3p”	0.986	0.958	1	0.275	22.9	−4.02 −3.25
“miR-21-5p + miR-320a-3р”	0.958	1	0.888	0.425	11.39	−0.66 −2.19
Placenta increta						
“Clusterin”	1	1	1	0.5	287.91	−372.61
“miR-21-5p + miR-320a-3p”	0.981	0.958	1	0.263	16.70	−1.25 −2.41
“miR-21-5p + miR-92a-3p + miR-320a-3р”	0.981	0.916	1	0.277	16.96	−1.24 −0.16 −2.34
“miR-21-5p”	0.958	1	0.933	0.73	12.35	−1.89
“miR-21-5p + miR-92a-3p”	0.958	1	0.933	0.545	15.59	−1.68 −1.25
Placenta percreta						
“miR-320a-3p”	1	1	1	0.5	828.41	−298.44

AUC—area under the curve; Sp—specificity; Se—sensitivity; cutoff—intersection point at which there is a balance between sensitivity and specificity; i and K—coefficients used in Equation (1).

## Data Availability

The NGS data for this study have been deposited in the European Nucleotide Archive (ENA) at EMBL-EBI under accession number PRJEB43546 (https://www.ebi.ac.uk/ena/browser/view/PRJEB43546), accessed on 9 March 2021.

## Supplementary Materials

The following are available online at https://www.mdpi.com/2075-1729/11/4/270/s1, Table S1: Deep sequencing of miRNAs from placenta tissue samples in N- and P-areas, Table S2: Logistic regression models parameters.
